# NeuroFusionNet: cross-modal modeling from brain activity to visual understanding

**DOI:** 10.3389/fncom.2025.1545971

**Published:** 2025-03-26

**Authors:** Kehan Lang, Jianwei Fang, Guangyao Su

**Affiliations:** ^1^School of Mathematical Sciences, Nankai University, Tianjin, China; ^2^China Unicom Software Research Institute, Beijing Economic and Technological Development Zone, Beijing, China

**Keywords:** cognitive computing, neuroscience, deep learning, machine vision, cross-modal fusion

## Abstract

In recent years, the integration of machine vision and neuroscience has provided a new perspective for deeply understanding visual information. This paper proposes an innovative deep learning model, NeuroFusionNet, designed to enhance the understanding of visual information by integrating fMRI signals with image features. Specifically, images are processed by a visual model to extract region-of-interest (ROI) features and contextual information, which are then encoded through fully connected layers. The fMRI signals are passed through 1D convolutional layers to extract features, effectively preserving spatial information and improving computational efficiency. Subsequently, the fMRI features are embedded into a 3D voxel representation to capture the brain's activity patterns in both spatial and temporal dimensions. To accurately model the brain's response to visual stimuli, this paper introduces a Mutli-scale fMRI Timeformer module, which processes fMRI signals at different scales to extract both fine details and global responses. To further optimize the model's performance, we introduce a novel loss function called the fMRI-guided loss. Experimental results show that NeuroFusionNet effectively integrates image and brain activity information, providing more precise and richer visual representations for machine vision systems, with broad potential applications.

## 1 Introduction

In recent years, computer vision technology has experienced rapid development, achieving significant progress, particularly in the field of cross-modal understanding (Allen et al., 2022; Anantharaman et al., 2018; Ning et al., 2024). Models such as CLIP and Diffusion have greatly enhanced the semantic understanding capabilities of vision systems by incorporating natural language as guidance (Sannidhan et al., 2023; Kodipalli et al., 2023). These models are not only capable of extracting structured features from images but also augment the semantic representation of visual scenes through textual input, enabling richer cross-modal semantic associations (Hao et al., 2024; Bao et al., 2021; Mann et al., 2020; Chen et al., 2019). However, such methods primarily rely on explicit mappings between visual and linguistic data, leaving challenges in terms of model depth and generalization, especially when modeling complex cognitive processes.

Functional magnetic resonance imaging (fMRI) captures the dynamic cognitive activities involved in recognition processes, offering a novel perspective for computer vision research (Chen et al., 2020a,b; Wang et al., 2025). For instance, Brain-Streams explores how modern latent diffusion models (LDMs) generate structurally and semantically coherent images through multi-modal guidance (text, vision, and image layouts) (Joo et al., 2024). By extracting text guidance from semantic regions and visual guidance from perceptual regions, it provides precise multi-modal information for the model. MindLDM proposes a new cross-subject visual reconstruction method, utilizing a masked autoencoder (MAE) to extract latent features from fMRI and mapping them to the CLIP text feature space (Guo et al., 2024). This is combined with a very deep variational autoencoder (VDVAE) to capture contour information from visual inputs, ultimately reconstructing visual stimuli via a latent diffusion model integrated with ControlNet. DREAM introduces a customized pathway to decode semantics, color, and depth cues from fMRI data, simulating the forward pathway from visual stimuli to fMRI recordings (Xia et al., 2024). It builds two reverse components to infer the associations and hierarchy of the human visual system. MindEye, on the other hand, focuses on preserving fine-grained image-specific information, improving the accuracy of image retrieval (Scotti et al., 2024). However, these methods predominantly rely on mapping fMRI signals to the CLIP feature space to model visual cognition, which leads to the loss of rich spatiotemporal information and cognitive depth, limiting their ability to comprehensively represent the brain's multi-dimensional functional characteristics.

A deeper understanding of brain function is expected to drive the development of novel deep neural networks. The cognitive processes in the human visual system are complex and multidimensional, containing rich spatiotemporal feature information that could provide more vivid references for artificial intelligence models (He et al., 2016). In studying how the human brain processes visual information, many researchers have found that different regions of the brain perform distinct tasks and collaboratively contribute to cognition, judgment, and decision-making (Chen et al., 2023). With a more comprehensive understanding of brain function, we can gain inspiration for designing deep neural networks that better align with human visual cognition. This will raise the bar for future machine vision systems, enabling models to process visual information at a level closer to human thinking.

This paper revisits an early key question in artificial intelligence: if human brain behavior could guide machine vision models, could it enhance the precision and generalization of visual understanding? The NeuroFusionNet model proposed in this paper aims to enhance the depth of visual understanding by integrating fMRI signals and visual features. First, the image is processed through a visual model to extract region-of-interest (ROI) features and contextual information, which are then encoded. Meanwhile, the fMRI signals are processed through 1D convolutional layers to extract brain activity features, preserving spatial information, and embedding them into a 3D voxel representation to capture spatiotemporal activity patterns. Next, a Mutli-scale fMRI Timeformer module is designed to process fMRI signals at different scales, extracting both detailed and global responses. Finally, cross-modal fusion is performed to combine the visual and fMRI features, forming a richer visual representation. To optimize model performance, an fMRI-guided loss function is introduced to further improve the accuracy of the model in visual tasks.

The contributions of this paper are as follows:

- The NeuroFusionNet model proposed in this paper successfully integrates fMRI signals and visual features, combining brain activity information with visual data to enhance the depth of visual understanding. This innovative approach overcomes the limitations of traditional methods that rely heavily on large amounts of manually labeled data, utilizing the cognitive processes of the brain to guide visual tasks, and improving the model's ability to generalize across diverse scenarios.
- To effectively capture the spatiotemporal patterns of brain activity, this paper designs a 3D voxel embedding module that retains the spatial information of the fMRI signals in high-dimensional feature representations and further extracts features through 1D convolution layers. Additionally, a Mutli-scale fMRI Timeformer module is employed to process fMRI signals at different scales, effectively reducing computational complexity while ensuring the extraction of multi-level, multi-scale features, thus enhancing the model's expressive power.
- This paper also introduces an innovative loss function to optimize the alignment between visual features and fMRI features, promoting cross-modal feature fusion. The model is able to optimize both the accuracy of visual tasks and the spatiotemporal features of brain activity, further enhancing visual understanding and brain activity modeling.

The structure of this paper is as follows: Section 2 introduces related work, Section 3 provides a detailed description of NeuroFusionNet, Section 4 presents the experiments, and the paper concludes with Section 5.

## 2 Related work

### 2.1 Vision-language foundation models

In recent years, significant progress has been made in cross-modal learning based on vision and language, particularly through pre-training on large-scale datasets, enabling models to perform exceptionally well on various vision-language tasks. CLIP (Contrastive Language-Image Pre-training) is one of the pioneering works in this field (Cox and Savoy, 2003; Cui and Liang, 2022; Ning et al., 2023; Ye et al., 2025). By leveraging contrastive learning and pre-training on hundreds of millions of image-text pairs, it has established a strong correlation between images and text, mapping both vision and language into the same feature space, which has greatly improved performance on multimodal tasks. Following closely, BLIP (Bootstrapping Language-Image Pre-training) enhanced the pre-training strategy and adopted a more efficient self-supervised learning approach to further improve the accuracy of vision-language understanding (Deng et al., 2009; Haxby et al., 2001; Ning et al., 2022; Bandyopadhyay et al., 2025). In terms of self-supervised training, MAE (Masked Autoencoders) successfully reduced the reliance on manually labeled data by masking parts of an image and training the model to recover the missing parts, thereby improving the model's representational capacity. Additionally, many methods have employed dual encoder architectures, such as ViLBERT and LXMERT (He et al., 2022). These models separately encode images and text, then pretrain them using contrastive learning or generative loss, optimizing the fusion of visual and linguistic information. ALIGN (A Large-scale Image and Noisy-text embedding) adopts a similar approach, training on image and noisy-text pairs to enhance the model's cross-modal learning ability (Devlin, 2018; Haynes and Rees, 2005). These methods have significantly advanced the development of vision-language models and provided new solutions for more complex visual understanding tasks.

In this paper, we use fMRI signals as supervisory signals to constrain the learning process of neural networks. Unlike traditional vision models that rely on large-scale labeled datasets, fMRI signals provide dynamic information about brain activity, revealing the spatiotemporal features involved in the visual cognitive process.

### 2.2 Decoding functional MRI

The application of functional magnetic resonance imaging (fMRI) in decoding visual information has made significant progress, with many studies attempting to reveal how the brain processes visual stimuli and performs cognitive tasks. Early works proposed that by analyzing the activity patterns in the visual cortex and their relationship with image categories, it was possible to link brain activity with specific object categories (e.g., faces, objects) (Duan et al., 2024; Wang et al., 2025; Screven et al., 2025). However, these methods have limitations when dealing with complex visual stimuli and individual differences. They are also sensitive to noise, which affects their generalizability across different individuals or scenarios (Dosovitskiy, 2020; Graves et al., 2016). To address these issues, some studies employed statistical learning methods such as Support Vector Machines (SVM), using large training datasets to improve decoding accuracy. However, these methods still face challenges related to their reliance on large datasets and their insufficient real-time decoding capabilities. Another approach used multivariate linear regression to successfully reconstruct simple visual stimuli from fMRI signals, but the performance was poor when handling complex images or dynamically changing visual stimuli, and the decoding speed was slow (Dwivedi et al., 2021; Gifford et al., 2023; Hu et al., 2025). To further improve decoding accuracy, researchers have proposed deep learning methods based on Convolutional Neural Networks (CNN) and Recurrent Neural Networks (RNN) (Gregor et al., 2015). These methods can automatically extract features from fMRI signals and capture the temporal changes in visual stimuli. While these methods have shown promising results, they still depend on large labeled datasets and have high computational costs, limiting their use in real-time decoding. In recent years, innovative methods based on Graph Neural Networks (GNN) and Transformers have been proposed, utilizing the connectivity between brain regions and self-attention mechanisms to further enhance decoding accuracy and spatiotemporal dependencies. However, the ability to handle high-dimensional data and noise remains a key challenge that needs further optimization (Fong et al., 2018).

These studies demonstrate the potential of fMRI signals in decoding visual information, but most methods still face challenges such as high model complexity, strong data dependence, and large individual differences, which limit their broad application in practice. Furthermore, it is worth noting that these methods generally do not leverage the features of regions of interest (ROIs) in the brain, which contain rich information about visual stimuli.

## 3 The proposed approach

### 3.1 The overall network

**Figure 1:**
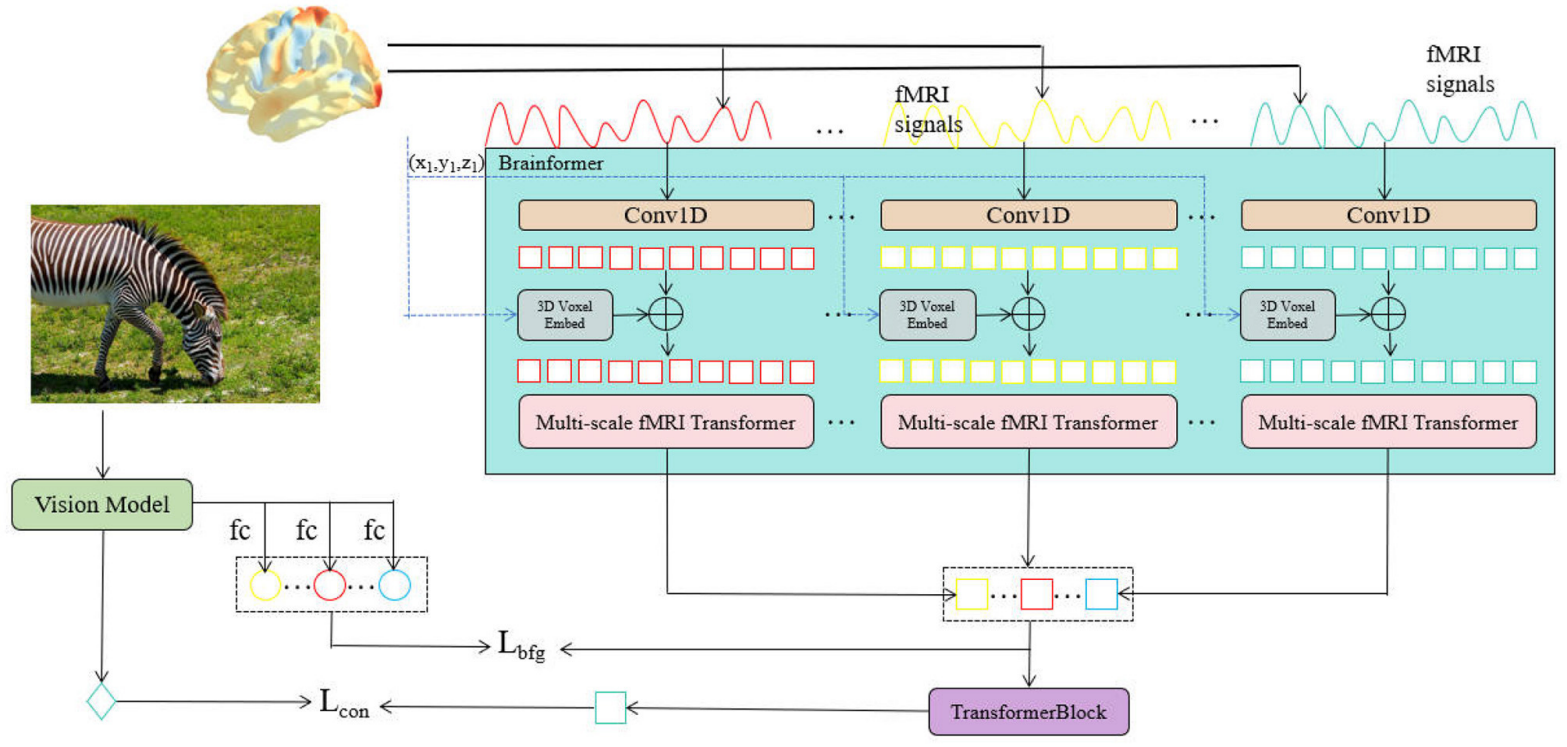
The overall network structure of NeuroFusionNet.

The proposed NeuroFusionNet, as shown in Figure 1, takes fMRI signals and images as input to achieve a deep understanding of visual information. Specifically, the image is processed through a visual model to extract visual region features (ROI features) and contextual features, which are further encoded through fully connected layers for downstream visual tasks. Meanwhile, the fMRI signals capture brain activity related to visual stimuli, and these signals are processed through 1D convolution layers for feature extraction. The features are then embedded into a 3D voxel representation to capture the brain's activity patterns across different spatial and temporal scales. Next, the Mutli-scale fMRI Timeformer module processes the fMRI signals at different scales, extracting both fine-grained and global responses of the brain to visual stimuli. The extracted fMRI features are then combined with the visual features generated by the visual model and input into the Transformer Block, where cross-modal feature fusion encoding takes place, forming a richer visual representation. To optimize the model's performance, two loss functions are designed: a guidance loss (*L*_*con*_) to align the fMRI features with the visual features, and another loss (*L*_*bfg*_) to further improve the accuracy of the visual model in visual tasks.

### 3.2 Brain 3D voxel embedding

In the Brain 3D Voxel Embedding module, 3D voxel embedding is used to represent the three-dimensional spatial information in the fMRI signals. Traditional methods that directly extract features from flattened signals often ignore the spatial structure of the fMRI signals, making it difficult to capture the complex relationships between brain regions. To address this issue, the module first maps the spatial coordinates of each voxel (*x*_*ik*_, *y*_*ik*_, *z*_*ik*_) to the feature space through a linear transformation, obtaining a high-dimensional feature representation:


$$v_{ik}=\text{Linear}\left(x_{ik},y_{ik},z_{ik}\right)$$

where *v*_*ik*_ is the high-dimensional feature representation of voxel *i* at the *k*-th time step, retaining the spatial location of each voxel to facilitate capturing its spatial structural information.

Next, the features of all voxels are concatenated to generate a 3D voxel embedding representation of the entire brain:


$$v\left(m_{k}\right)=\text{concat}\left(v_{0k},v_{1k},\ldots ,v_{N-1k}\right)$$

where *v*(*m*_*k*_) is the joint feature representation of all voxels at the *k*-th time step, capturing the spatial correlations between the voxels through concatenation. This joint representation provides multi-dimensional information for subsequent convolution operations.

Finally, the embedding is further processed through a 1D convolution operation, combining the 3D voxel embedding information to obtain a compressed feature representation:


$$r_{k}=\text{Conv1D}\left(m_{k}\right)+v\left(m_{k}\right)$$

where *r*_*k*_ is the final voxel feature representation at the *k*-th time step, which combines the convolution features from *m*_*k*_ and the 3D spatial information from *v*(*m*_*k*_). This representation fuses convolution and voxel embedding information through addition, retaining the spatial structure and activity patterns of the fMRI signals.

### 3.3 Mutli-scale fMRI Timeformer

**Figure 2:**
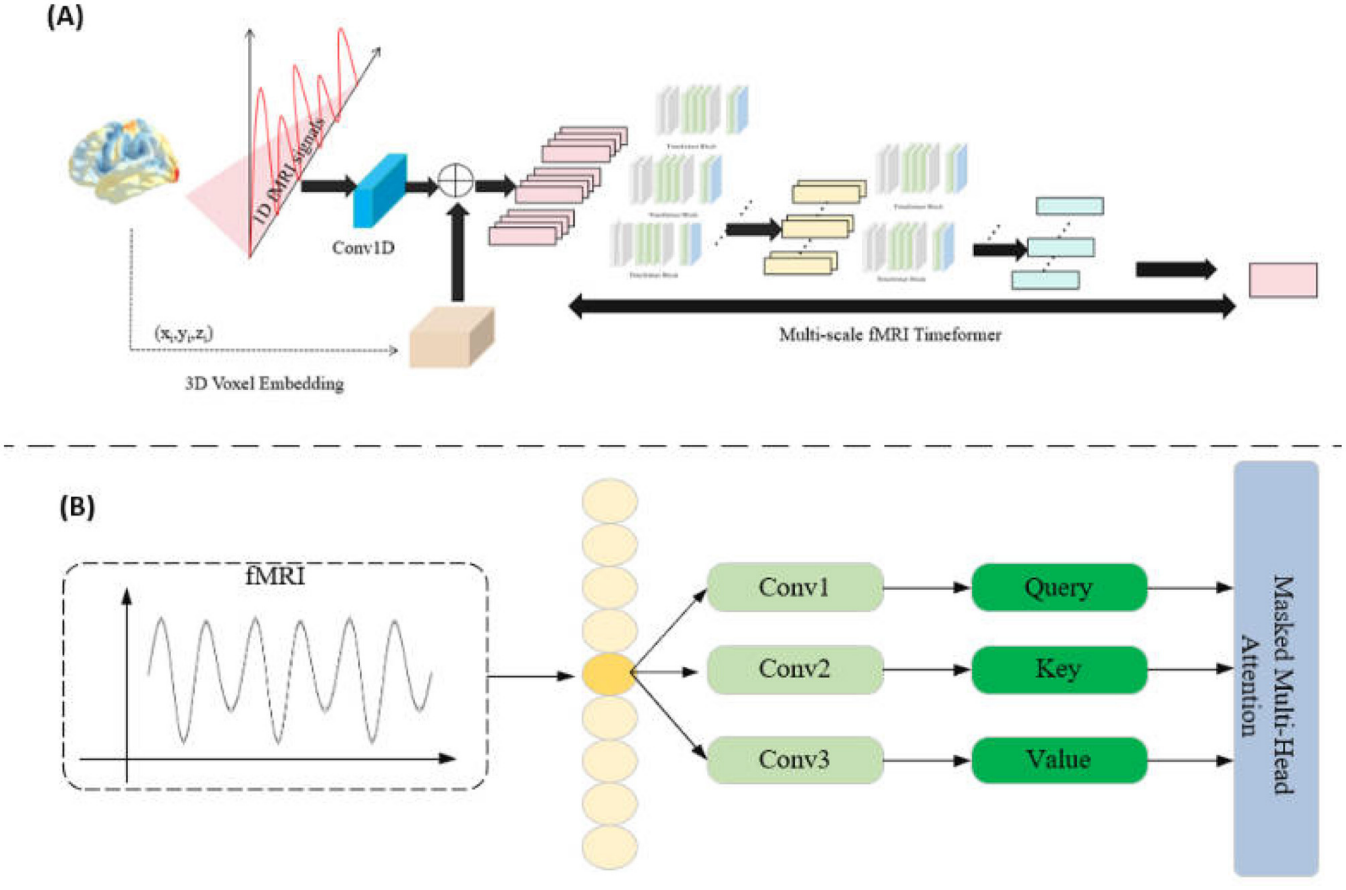
**(A)** Multi-scale fMRI Timeformer overall network structure. **(B)** Timeformer block network structure.

In the Mutli-scale fMRI Timeformer module, the primary goal is to address the computational complexity issue that arises when traditional Transformers process high-dimensional fMRI signals. To reduce the computational demand, this module employs a slicing window approach, allowing the model to learn signal patterns within local regions while maintaining computational efficiency. The Transformer block is shown in Figure 2.

Specifically, for each fMRI signal representation *r*_*k*_, we apply a slicing window of width *w* to process the signal in chunks. This slicing window starts from the beginning of the signal *r*_*k*_ and slides with a stride *s* until the entire signal sequence is covered. For each step *i*, we obtain a slice *q*_*ik*_, which can be represented as:


$$q_{ik}=r_{k}\left[i\leq s:i\leq s+w\right]$$

where 0 ≤ *i* ≤ *n* and $s=\frac{N_{k}}{s_{0}}$, with *N*_*k*_ representing the total length of the signal *r*_*k*_.

Each slice *q*_*ik*_ is fed into the Transformer submodule (*transBlock*) to learn patterns within the slice and obtain feature representations:


$$q_{ik}=transBlock_{k}\left(q_{ik}\right)$$

In this way, the Mutli-scale fMRI Timeformer captures features of the fMRI signal in local regions, thereby reducing computational complexity. Meanwhile, all the slices *q*_*ik*_ are collected as input to the next layer of the Transformer.

In the multi-layer Transformer, each layer uses the feature representations obtained from the previous layer as input, progressively enhancing the model's expressive power. Ultimately, in the last Transformer block, we obtain the final feature representation of the signal. This final feature representation ${\bar{q}}_{k}$ integrates multi-level information from the fMRI signal, capturing multi-scale features within the signal. This hierarchical, multi-scale processing approach effectively alleviates the computational burden while ensuring that the model can extract rich spatiotemporal patterns from the signal.

### 3.4 Loss function

In this paper, we design a composite loss function *L* to optimize the alignment between visual features and fMRI features, achieved by introducing contrastive loss *L*_con_ and fMRI-guided loss *L*_bfg_.

#### 3.4.1 Contrastive loss

The contrastive loss *L*_con_ is primarily used to align visual features *p*_*i*_ and fMRI features *q*_*i*_, ensuring their similarity within the same feature space. This loss function enhances the similarity of matching feature pairs and reduces the similarity of non-matching feature pairs through contrastive learning. Specifically, for each sample *i*, the contrastive loss is defined as:


$$\begin{array}{l} L_{\text{con}}=-\frac{1}{N}\overset{N}{\underset{i=1}{\sum }}\log\frac{\exp\left(p_{i}⊗q_{i}/\sigma \right)}{\sum_{j=1}^{N}\exp\left(p_{i}⊗q_{j}/\sigma \right)}\\ \;-\frac{1}{N}\overset{N}{\underset{i=1}{\sum }}\log\frac{\exp\left(q_{i}⊗p_{i}/\sigma \right)}{\sum_{j=1}^{N}\exp\left(q_{i}⊗p_{j}/\sigma \right)} \end{array}$$

where *p*_*i*_ represents the visual feature of the *i*-th sample; *q*_*i*_ represents the fMRI feature of the *i*-th sample; ⊗ denotes the dot product operation used to calculate the similarity between features; σ is a temperature parameter that adjusts the smoothness of the contrast; *N* is the total number of samples.

By maximizing the similarity of matching feature pairs (e.g., *p*_*i*_ and *q*_*i*_) while minimizing the similarity of non-matching feature pairs, the model learns to align visual and fMRI features in the same feature space. This contrastive learning mechanism enables the visual model to effectively leverage information from the fMRI features, improving the performance of visual tasks.

#### 3.4.2 fMRI-guided loss

The fMRI-guided loss *L*_bfg_ is designed to align the local features of the vision model with the ROI features extracted from fMRI data, mimicking how the brain processes visual stimuli. This alignment mechanism maximizes the similarity between the vision model's local features and the corresponding brain region features, while minimizing confusion with features from other regions. The loss function is defined as:


$$L_{\text{bfg}}=-\frac{1}{N_{r}}\overset{N}{\underset{i=1}{\sum }}\overset{N_{r}}{\underset{k=1}{\sum }}\log\frac{\exp\left({\bar{p}}_{ik}⊗{\bar{q}}_{ik}\right)}{\sum_{g=1}^{N_{r}}\exp\left({\bar{p}}_{ik}⊗{\bar{q}}_{ig}\right)}$$

where: ${\bar{p}}_{ik}$: The *k*-th local feature extracted from the *i*-th sample by the vision model. This feature may represent specific parts of the image, such as an object, background, or scene region. ${\bar{q}}_{ik}$: The feature extracted from the *k*-th ROI of the brain for the *i*-th sample, representing the brain's response to the visual stimulus. For example, the floc-bodies region may correspond to object perception, while the floc-faces region may correspond to facial recognition. *N*_*r*_: The total number of ROIs in the brain, such as floc-bodies, floc-faces, etc. ⊗: The dot product operation, used to measure similarity between two feature vectors.

The process of the fMRI-guided loss *L*_bfg_ involves three key steps. First, for each input image, the vision model extracts local features ${\bar{p}}_{ik}$ through convolution and pooling layers, which are then projected into the same dimensional space as the brain's ROI features ${\bar{q}}_{ik}$ using a linear transformation. Simultaneously, fMRI data is divided into multiple ROIs, and features ${\bar{q}}_{ik}$ are extracted from each region to represent the brain's functional and stimulus response characteristics. Next, the similarity between the vision model's local features ${\bar{p}}_{ik}$ and the corresponding brain ROI features ${\bar{q}}_{ik}$ is maximized, ensuring alignment between features, such as facial features aligning well with floc-faces. At the same time, the similarity between ${\bar{p}}_{ik}$ and unrelated ROI features ${\bar{q}}_{ig}$ (*g*≠*k*) is minimized to prevent misalignment, such as background features aligning with object-related brain regions. Finally, the loss function is iteratively optimized during training, progressively guiding the vision model to learn the localized perception characteristics of the brain.

By incorporating the fMRI-guided loss *L*_bfg_, the vision model can capture the regional characteristics of brain activity in response to visual stimuli. This approach enables the model to align not only global features (aligned by contrastive loss) but also local features, effectively emulating how the brain processes objects, backgrounds, and other localized contents within images. As a result, the model achieves improved performance and interpretability in visual tasks.

#### 3.4.3 Overall loss

The final total loss function is:


$$L=\lambda_{\text{con}}L_{\text{con}}+\lambda_{\text{bfg}}L_{\text{bfg}}$$

where, λ_con_ and λ_bfg_ are hyperparameters used to control the relative weights of the contrastive loss and the guided loss. Through this combination, the model is able to simultaneously optimize the similarity of aligned features and the spatiotemporal mapping relationship when processing fMRI signals and visual tasks, thereby improving visual understanding and brain activity modeling.

## 4 Experiments

### 4.1 Datasets

The dataset used in this study is the Natural Scenes Dataset (NSD) (Allen et al., 2022), a large-scale fMRI dataset collected using a 7T ultra-high field MRI scanner at the Center for Magnetic Resonance Research (cMRR) at the University of Minnesota. The dataset includes high-resolution whole-brain fMRI data (1.8 mm isotropic resolution, 1.6-second sampling rate) from 8 healthy adult subjects. During 30 to 40 scanning sessions, each subject observed thousands of color natural scene images while participating in a continuous visual task. The dataset is designed to investigate the neural responses of the brain to natural scenes and provides high-quality functional imaging data for in-depth analysis of the brain's visual processing mechanisms.

### 4.2 Model training

During the training process, we used data from seven participants in the Natural Scenes Dataset (NSD), with the data of one participant reserved for testing. To ensure consistency of the input data, all images were preprocessed and resized to a uniform size of 224 × 224 pixels before training. For the processing of fMRI signals, the brain was divided into six regions of interest (ROIs) to facilitate targeted analysis. In the model architecture, we employed 1D convolutional layers (Conv1D) to process the fMRI data, with a kernel size and stride set to 32 and 16, respectively. Subsequently, the model incorporated a multi-scale fMRI Transformer module, configured with a window size of *w* = 64, a stride of *s* = 32, and *h* = 2 Transformer layers to capture the multi-scale features of fMRI signals. During training, the initial learning rate was set to 0.0001, and the model was trained for a total of 100 epochs. This design and parameter configuration provide robust support for standardized input, feature extraction, and multi-scale signal modeling, ensuring efficient feature learning and stable performance of the model.

### 4.3 Experimental environment

The experiments were conducted on a high-performance computing platform equipped with 2 NVIDIA A100 GPUs, a 64-core Intel Xeon processor, and 128 GB of memory, ensuring efficient data processing and deep learning model training for large-scale tasks. All experiments were run in the Ubuntu 20.04 LTS operating system environment, using PyTorch 1.10 as the deep learning framework, with GPU acceleration provided by CUDA 11.3 and cuDNN 8.2. Additionally, Python 3.8 was used for writing experimental scripts, and dependencies and environments were managed through Anaconda virtual environments. For downstream tasks such as object detection and image segmentation, the MMDetection framework was utilized. MMDetection offers a modular and flexible platform for developing state-of-the-art object detection models, with a rich set of pre-trained models and task-specific optimization tools.

### 4.4 Evaluation metrics

In this paper, we use the following metrics to evaluate the performance of NeuroFusionNet. AP^box^, ${\text{AP}}_{50}^{\text{box}}$, and ${\text{AP}}_{75}^{\text{box}}$ are the core evaluation metrics for object detection tasks. Among them, AP^box^ represents the average precision across multiple IoU (Intersection over Union) thresholds and is used to assess the overall detection performance of the model. The formula for AP is defined as:


$$\text{AP}=\frac{1}{N}\overset{N}{\underset{i=1}{\sum }}{\text{Precision}}_{i}\times \Delta {\text{Recall}}_{i}$$

where Precision_*i*_ and Recall_*i*_ represent the precision and recall at the *i*-th point, respectively, and ΔRecall_*i*_ represents the recall increment between adjacent points. ${\text{AP}}_{50}^{\text{box}}$ refers to the average precision at an IoU threshold of 0.5, suitable for evaluating detection performance under relaxed conditions. ${\text{AP}}_{75}^{\text{box}}$, on the other hand, represents the average precision at an IoU threshold of 0.75, which reflects the model's ability to meet stricter detection requirements.

mIoU (Mean Intersection over Union) is an important metric for evaluating the performance of instance segmentation tasks. It represents the average IoU across all categories and reflects the overlap between the predicted segmentation results and the ground truth regions. The definition of IoU is given by the ratio of the overlap area to the union area between the predicted and ground truth regions:


$$\text{IoU}=\frac{\text{Area of Overlap}}{\text{Area of Union}}$$

The mean IoU across all categories is defined as:


$$\text{mIoU}=\frac{1}{C}\overset{C}{\underset{c=1}{\sum }}{\text{IoU}}_{c}$$

where *C* represents the total number of categories, and IoU_*c*_ is the IoU value for the *c*-th category. mIoU measures the model's segmentation capability across multiple categories and is a crucial standard for evaluating segmentation performance. Using these metrics, we comprehensively assess the performance of NeuroFusionNet in object detection and instance segmentation tasks.

### 4.5 Experimental details

In this paper, we adopt Swin-S and ConvNext-S as the backbone networks for image processing, and use the Mask R-CNN framework to improve performance and efficiency through the MMDetection framework. Specifically, we employ three pre-training strategies: Random init, CLIP, and NeuroFusionNet, to optimize visual feature extraction and cross-modal fusion.

#### 4.5.1 Random init

Random init refers to training the network from random initialization. In this process, the image is directly fed into the backbone network (such as Swin-S or ConvNext-S), and training starts from scratch without using any pre-trained features. This is the most basic training method, which can test the model's performance without prior knowledge. For Swin-S or ConvNext-S, training starts from random initialization:


$$v_{\text{init}}={\text{Swin-S}}_{\text{visual}}\left(I\right)\;\text{or}\;v_{\text{init}}={\text{ConvNext-S}}_{\text{visual}}\left(I\right)$$

where *I* is the input image and *v*_init_ is the initial visual feature.

#### 4.5.2 CLIP

CLIP is a multi-modal contrastive learning model that maps images and texts to the same feature space through large-scale image-text pair training. First, the image is input into CLIP, and the visual encoder in CLIP extracts the feature vector of the image. The formula for feature extraction:


$$v_{\text{clip}}={\text{CLIP}}_{\text{visual}}\left(I\right)$$

where *I* is the input image, and *v*_clip_ is the visual feature vector extracted from CLIP.

Next, the visual feature *v*_clip_ extracted by CLIP is used as initialization and transferred to Swin-S or ConvNext-S as the visual feature extraction part of the backbone network. The formula for transfer:


$$v_{\text{init}}={\text{Swin-S}}_{\text{visual}}\left(I\right)\;\text{or}\;v_{\text{init}}={\text{ConvNext-S}}_{\text{visual}}\left(I\right)$$

In this way, Swin-S or ConvNext-S gains the pre-trained advantage provided by CLIP when processing images, allowing the network to better understand image content, especially when performing object detection and instance segmentation.

#### 4.5.3 NeuroFusionNet

NeuroFusionNet is a model that performs pre-training by integrating fMRI signals and images, similar to the idea behind CLIP. First, the image is input into NeuroFusionNet, where its visual encoder extracts the feature vector of the image. The feature extraction formula is as follows:


$$v_{\text{NeuroFusionNet}}={\text{NeuroFusionNet}}_{\text{visual}}\left(I\right)$$

where *I* is the input image, and *v*_NeuroFusionNet_ is the visual feature vector extracted by NeuroFusionNet.

Next, the visual feature *v*_NeuroFusionNet_ extracted by NeuroFusionNet is used as the initialization and transferred to the Swin-S or ConvNext-S network as part of the backbone network for visual feature extraction. The transfer formula is as follows:


$$v_{\text{init}}={\text{Swin-S}}_{\text{visual}}\left(I\right)\;\text{or}\;v_{\text{init}}={\text{ConvNext-S}}_{\text{visual}}\left(I\right)$$

In this way, Swin-S or ConvNext-S benefits from the pre-trained advantage provided by NeuroFusionNet, which integrates both visual information and brain activity. This allows the network not only to extract visual features from the image but also to incorporate the brain's response patterns to these visual stimuli, further improving the precision of image understanding.

### 4.6 Object detection

Experiments on object detection and instance segmentation were conducted on the COCO 2017 dataset. COCO 2017 is a large-scale dataset with numerous annotated images, commonly used to evaluate the performance of computer vision models in complex scenes.

The experimental results, as shown in Table 1, demonstrate that NeuroFusionNet significantly outperforms random initialization and CLIP pre-training methods across several key evaluation metrics, particularly in AP^box^, ${\text{AP}}_{50}^{\text{box}}$, and ${\text{AP}}_{75}^{\text{box}}$ for object detection and instance segmentation tasks.

**Table 1:** Performance of object detection and instance segmentation on the COCO dataset.

	**Backbone**	**Pretrain**	**AP^box^**	** ${\text{AP}}_{50}^{\text{box}}$ **	** ${\text{AP}}_{75}^{\text{box}}$ **
**(a) Object detection**	Swin-S	Random init	41.4 (±0.3)	63.5 (±0.6)	45.6 (±0.5)
	Swin-S	CLIP (Hafner et al., 2021)	42.0 (±0.2)	64.7 (±0.5)	46.1 (±0.4)
	Swin-S	NeuroFusionNet	43.7 (±0.3)	65.9 (±0.6)	47.5 (±0.4)
	ConvNext-S	Random init	42.7 (±0.2)	65.5 (±0.7)	46.0 (±0.3)
	ConvNext-S	CLIP (Hafner et al., 2021)	42.9 (±0.2)	66.2 (±0.7)	46.3 (±0.5)
	ConvNext-S	NeuroFusionNet	45.2 (±0.2)	68.3 (±0.3)	50.1 (±0.3)
	Backbone	Pre-train	AP^segm^	${\text{AP}}_{50}^{\text{segm}}$	${\text{AP}}_{75}^{\text{segm}}$
**(b) Semantic segmentation**	Swin-S	Random init	38.5 (±0.3)	60.9 (±0.4)	41.4 (±0.3)
	Swin-S	CLIP (Hafner et al., 2021)	39.3 (±0.3)	61.4 (±0.4)	42.0 (±0.5)
	Swin-S	NeuroFusionNet	43.2 (±0.3)	64.1 (±0.6)	43.4 (±0.3)
	ConvNext-S	Random init	39.9 (±0.5)	62.3 (±0.3)	42.1 (±0.4)
	ConvNext-S	CLIP (Hafner et al., 2021)	40.9 (±0.4)	63.1 (±0.4)	43.1 (±0.5)
	ConvNext-S	NeuroFusionNet	44.1 (±0.3)	65.1 (±0.4)	45.4 (±0.5)

Values represent mean performance across multiple runs, with 95% confidence intervals indicated in parentheses.

In the object detection task, for the Swin-S backbone, NeuroFusionNet achieves an AP^box^ of 43.7 (±0.3), representing improvements of 2.3 percentage points and 1.7 percentage points over random initialization and CLIP, respectively. For the more specific metrics, ${\text{AP}}_{50}^{\text{box}}$ and ${\text{AP}}_{75}^{\text{box}}$, NeuroFusionNet achieves 65.9 (±0.6) and 47.5 (±0.4), which correspond to increases of 2.4 and 1.9 percentage points compared to random initialization, and 1.2 and 1.4 percentage points compared to CLIP. For the ConvNext-S backbone, NeuroFusionNet demonstrates even greater performance, achieving an AP^box^ of 45.2 (±0.2), which is 2.5 and 2.3 percentage points higher than random initialization and CLIP, respectively. Furthermore, NeuroFusionNet achieves 68.3 (±0.3) in ${\text{AP}}_{50}^{\text{box}}$ and 50.1 (±0.3) in ${\text{AP}}_{75}^{\text{box}}$, surpassing random initialization by 2.8 and 4.1 percentage points, and CLIP by 2.1 and 3.8 percentage points, respectively.

In the instance segmentation task, NeuroFusionNet also demonstrates outstanding performance. For the Swin-S backbone, NeuroFusionNet achieves an AP^box^ of 43.2 (±0.3), showing improvements of 4.7 and 3.9 percentage points over random initialization and CLIP, respectively. On the ${\text{AP}}_{50}^{\text{box}}$ and ${\text{AP}}_{75}^{\text{box}}$ metrics, NeuroFusionNet achieves 64.1 (±0.6) and 43.4 (±0.3), representing increases of 3.2 and 2.0 percentage points compared to random initialization, and 2.7 and 1.4 percentage points compared to CLIP. For the ConvNext-S backbone, NeuroFusionNet achieves an AP^box^ of 44.1 (±0.3), outperforming random initialization and CLIP by 4.2 and 3.2 percentage points, respectively. In terms of ${\text{AP}}_{50}^{\text{box}}$ and ${\text{AP}}_{75}^{\text{box}}$, NeuroFusionNet achieves 65.1 (±0.4) and 45.4 (±0.5), with improvements of 2.8 and 3.3 percentage points over random initialization, and 2.0 and 2.3 percentage points over CLIP, respectively.

### 4.7 Semantic segmentation

Experiments on semantic segmentation were conducted on the ADE20K dataset. ADE20K is a standard dataset with 150 semantic categories, consisting of 25,000 images: 20,000 for training, 2,000 for validation, and 3,000 for testing. This dataset covers a wide range of scenes and object categories, making it an important benchmark for evaluating semantic segmentation models.

As shown in Table 2, in the experiments on semantic segmentation on the ADE20K dataset and brain activity prediction on the NSD dataset, NeuroFusionNet showed significant improvements compared to other pre-training methods (random initialization and CLIP pre-training). On the mIoU evaluation metric for the ADE20K dataset, NeuroFusionNet improved by about 3.4 percentage points for the Swin-S backbone network compared to random initialization, and by about 1.5 percentage points compared to CLIP pre-training. For the ConvNext-S backbone network, NeuroFusionNet improved by about 3.7 percentage points compared to random initialization, and by about 1.6 percentage points compared to CLIP pre-training.

**Table 2:** Results of semantic segmentation on ADE20K and brain activities response prediction on NSD.

**Backbone**	**Pre-train**	**ADE20K mIoU**	**NSD PCC**
Swin-S	Random init	38.38 (±0.2)	40.40 (±0.4)
	CLIP (Hafner et al., 2021)	40.27 (±0.3)	41.26 (±0.3)
	NeuroFusionNet	41.76 (±0.3)	44.64 (±0.2)
ConvNext-S	Random init	39.23 (±0.3)	54.21 (±0.3)
	CLIP (Hafner et al., 2021)	41.27 (±0.4)	55.71 (±0.2)
	NeuroFusionNet	42.92 (±0.3)	57.43 (±0.4)

Values represent mean performance across multiple runs, with 95% confidence intervals indicated in parentheses.

In the brain activity prediction task on the NSD dataset, NeuroFusionNet also performed excellently. For the Swin-S backbone network, NeuroFusionNet improved by about 4.2 percentage points on the PCC evaluation metric compared to random initialization, and by about 3.4 percentage points compared to CLIP pre-training. For the ConvNext-S backbone network, NeuroFusionNet improved by about 3.2 percentage points compared to random initialization, and by about 1.7 percentage points compared to CLIP pre-training. These results indicate that NeuroFusionNet has stronger feature extraction capability in complex visual and brain neural activity prediction tasks, effectively improving the model's performance and generalization ability.

### 4.8 Ablation studies

The ablation studies in this paper evaluate the impact of different settings on the model's performance, validating the effectiveness of each module and hyperparameter. As shown in Table 3, the brain 3D voxel embedding, brain fMRI guidance loss, and the hyperparameter optimization of the Mutli-scale fMRI Timeformer significantly improved the model's performance, achieving the best results, particularly in visual tasks and brain activity prediction tasks.

**Table 3:** Performance on various settings.

	**COCO**	**COCO**	**ADE20K**	**NSD**
	**AP^box^**	**AP^segm^**	**mIoU**	**PCC**
pos embed	41.8	41.7	41.73	56.18
3D voxel embed	**45.2**	**44.1**	**42.92**	**57.43**
w/o Brain fMRI guidance loss	42.6	40.6	41.81	56.23
w/ Brain fMRI guidance loss	**45.2**	**44.1**	**42.92**	**57.43**
*w* = 128, *s* = 64	41.8	41.2	40.91	55.81
*w* = 128, *s* = 32	42.3	41.7	41.41	56.20
*w* = 64, *s* = 32	**45.2**	**44.1**	**42.92**	**57.43**
# subjects = 1	42.5	39.4	39.16	54.51
# subjects = 3	42.9	39.7	39.36	54.52
# subjects = 5	43.3	40.5	41.81	56.22
# subjects = 7	**45.2**	**44.1**	**42.92**	**57.43**

Bold value represents the best results.

#### 4.8.1 Brain 3D voxel embedding

We evaluated the performance differences between using brain 3D voxel embedding and traditional position embedding across various tasks. The results show that the model with 3D voxel embedding significantly outperformed the position embedding model in all tasks, particularly in object detection and instance segmentation, where both AP^box^ and AP^segm^ showed substantial improvements. Specifically, the 3D voxel embedding achieved a 3.4 percentage point increase in AP^box^ on the COCO dataset compared to position embedding, and a 2.4 percentage point improvement in AP^segm^ for instance segmentation tasks. Additionally, 3D voxel embedding also significantly improved mIoU and PCC metrics on the ADE20K and NSD datasets.

#### 4.8.2 fMRI-guided loss

To evaluate the impact of fMRI-guided Loss on model performance, we conducted a comparison experiment with and without the guidance loss. The results show that introducing brain fMRI guidance loss significantly improved the model's performance, especially in visual tasks. In the absence of fMRI guidance loss, the model's AP^box^ and AP^segm^ on the COCO and ADE20K datasets were slightly lower compared to the model with fMRI guidance loss. Specifically, after adding the guidance loss, the AP^box^ on the COCO dataset improved by approximately 2.6 percentage points, and the mIoU on the ADE20K dataset improved by approximately 1.1 percentage points.

#### 4.8.3 Hyper-parameters for mutli-scale fMRI timeformer

We also investigated the impact of hyperparameter configurations for the Mutli-scale fMRI Timeformer module on model performance. By adjusting the window size (*w*) and stride (*s*), we found that the model achieved the best performance across all tasks when the window size was 64 and the stride was 32. This configuration performed excellently across all evaluation metrics on the COCO, ADE20K, and NSD datasets, with AP^box^ and AP^segm^ reaching 45.2 and 44.1, respectively, mIoU at 42.92, and PCC at 57.43.

#### 4.8.4 Performance on different amounts of data

Finally, we also examined the impact of different amounts of data on model performance. By increasing the number of samples, we found that as the sample size grew, the model's performance gradually improved, particularly in visual tasks, where the improvement was especially significant. When using only one subject, the model performed poorly on the ADE20K and COCO datasets. However, as more subjects were included, the model's performance showed a substantial improvement. When using seven subjects, the model achieved optimal performance across all datasets.

### 4.9 Visual analysis

**Figure 3:**
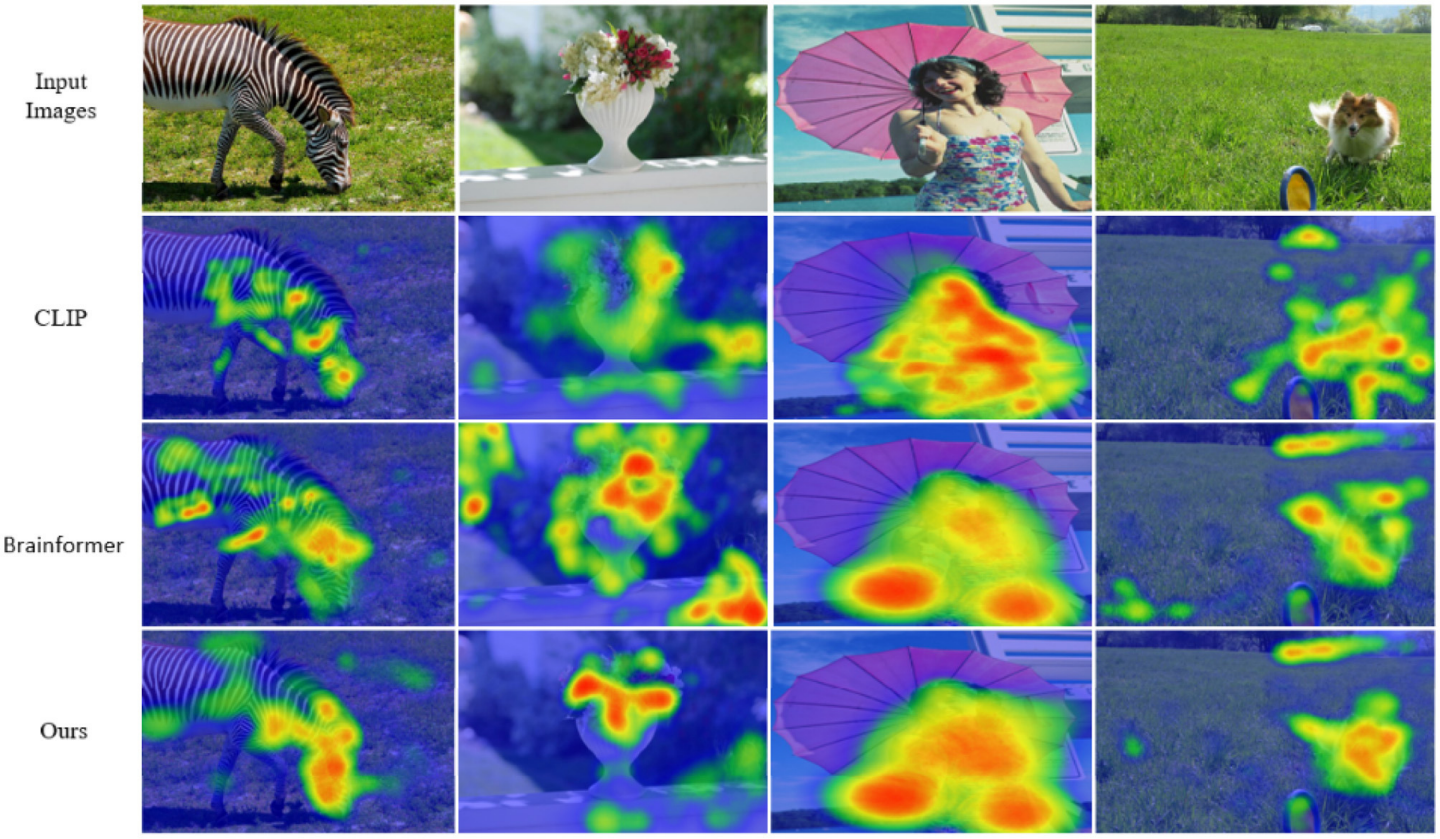
Visual attention with respect to fMRI signals.

As shown in Figure 3, we used Grad-CAM to perform feature visualization for NeuroFusionNet, CLIP, and Brainformer. Compared to CLIP and Brainformer, NeuroFusionNet demonstrates a more comprehensive and precise focus on semantically relevant regions. This indicates that NeuroFusionNet exhibits superior ability in aligning visual features with cognitive processes, effectively capturing important information from both visual stimuli and fMRI data.

### 4.10 Limitations and future works

One of the limitations of this work lies in the applicability of the dataset. Although the NSD dataset is representative in the field of neuroscience and is suitable for brain activity prediction tasks, its sample size and subject diversity are relatively limited. This may affect the model's performance in more complex scenarios. The dataset primarily comes from a specific group, and the sample size is insufficient to comprehensively represent different brain activity patterns. Therefore, the generalization ability of NeuroFusionNet across various subject groups and real-world applications still needs further validation. Future work could incorporate more diverse and representative datasets, particularly in clinical medicine or other application fields, to better assess the model's effectiveness and robustness.

Additionally, cross-modal data fusion remains a challenge for the model. Although NeuroFusionNet has shown promising results in both visual tasks and brain activity prediction, when integrating visual information and fMRI signals, the model may be affected by noise and data inconsistencies. The differences in spatiotemporal scales between the modalities make it difficult for the model to fully capture the deep relationships between them in some cases. This challenge could limit the model's performance in more complex tasks. Future research could explore more robust and efficient data fusion methods, such as adaptive weighting mechanisms or Generative Adversarial Networks (GANs), to better address inconsistencies and noise in cross-modal data fusion, thereby enhancing the model's stability and predictive accuracy.

### 4.11 Conclusions

This paper presents the NeuroFusionNet model, which aims to enhance the performance of visual tasks and brain activity prediction tasks through cross-modal data fusion. By combining image features with brain fMRI signals, the model is capable of simultaneously capturing visual information and brain responses, providing new insights and approaches for research in visual cognition and neuroscience. Experimental results demonstrate that NeuroFusionNet outperforms traditional vision models and other pre-training methods across multiple datasets, particularly in object detection, instance segmentation, and brain activity prediction tasks, showing significant performance improvements. These findings offer strong support and important references for future multimodal learning methods and neuroscience research.

## Data Availability

The original contributions presented in the study are included in the article/supplementary material, further inquiries can be directed to the corresponding author.
